# U2 snRNP Is Required for Expression of the 3′ End of Genes

**DOI:** 10.1371/journal.pone.0098015

**Published:** 2014-05-20

**Authors:** Mitsunori Koga, Takayuki Satoh, Ichiro Takasaki, Yumi Kawamura, Minoru Yoshida, Daisuke Kaida

**Affiliations:** 1 Frontier Research Core for Life Sciences, University of Toyama, Toyama, Japan; 2 Division of Molecular Genetics Research, Life Science Research Center, University of Toyama, Toyama, Japan; 3 Molecular Ligand Target Research Team, RIKEN Center for Sustainable Resource Science, Wako, Saitama, Japan; 4 Chemical Genetics Laboratory, RIKEN, Wako, Saitama, Japan; 5 JST, CREST, Kawaguchi, Saitama, Japan; INSERM, France

## Abstract

Pre-mRNA in eukaryotes is subjected to mRNA processing, which includes capping, polyadenylation, and splicing. Transcription and mRNA processing are coupled, and this coupling stimulates mRNA processing; however, the effects of mRNA processing on transcription are not fully understood. In this study, we found that inhibition of U2 snRNP by a splicing inhibitor, spliceostatin A (SSA), or by an antisense oligonucleotide to U2 snRNA, caused gene-specific 3′-end down-regulation. Removal of SSA from the culture media restored expression of the 3′ ends of genes, suggesting that U2 snRNP is required for expression of the 3′ end of genes. Finally, we found that SSA treatment caused accumulation of Pol II near the 5′ end of 3′-end down regulated genes, such as *CDK6*, *SMEK2* and *EGFR*, indicating that SSA treatment led to transcription elongation arrest on these genes. These findings suggest that U2 snRNP is important for production of full length mRNA probably through regulation of transcription elongation, and that a novel checkpoint mechanism prevents pre-mRNA from accumulating as a result of splicing deficiencies, and thereby prevents production of aberrant proteins that might be translated from pre-mRNAs through the arrest of transcription elongation.

## Introduction

In eukaryotes, nascent transcripts (precursor messenger RNAs; pre-mRNAs) are subjected to mRNA processing on the way to becoming mature mRNAs that can serve as templates for translation. Processing reactions include capping at the 5′ end, cleavage and polyadenylation at the 3′ end, and intron removal by splicing [1], [2], [3]. Such mRNA processing steps are critical for efficient gene expression and the integrity of the transcriptome; hence, aberrations in mRNA processing may perturb gene expression and interfere with vital cellular functions. For instance, because most eukaryotic genes contain intervening sequences (introns) that harbor numerous termination codons, the translation of intron-containing pre-mRNAs that accumulate as a result of splicing deficiencies may result in the production of truncated and possibly deleterious proteins [4]. To execute mRNA processing reactions precisely and efficiently, cells take advantage of the coupling between transcription and mRNA processing [5], [6]. When aberrant mRNA processing occurs, cells prevent the production of aberrant proteins from pre-mRNAs via several quality-control mechanisms, including pre-mRNA degradation, nonsense-mediated decay (NMD), and tethering of pre-mRNAs in the nucleus [7], [8], [9], [10], [11], [12].

The central player in mRNA splicing is the spliceosome, a macromolecular ribonucleoprotein complex. The spliceosome consists of five small ribonucleoprotein particles (snRNPs): U1, U2, U4, U5, and U6 [13]. Each snRNP contains a small nuclear RNA (snRNA) molecule, and the snRNAs play critical roles in the formation of the spliceosome. U1 snRNP recognizes its binding sequences on pre-mRNAs through RNA–RNA interactions between 5′ splice sites and U1 snRNA. U2 snRNA also forms RNA–RNA interactions with branch-point sequences. After the binding of the U4/U6•U5 tri-snRNP complex, conformational changes occur, resulting in formation of a catalytically active spliceosome. Two subsequent transesterification reactions excise the intron sequence and join the adjacent exons. The recent discovery of potent splicing inhibitors and the development of new technologies have allowed splicing activity to be controlled, thereby enabling investigation of the detailed mechanism of splicing and the interconnections between splicing and transcription [4], [14], [15]. One such inhibitor, spliceostatin A (SSA), is a methyl-ketal derivative of FR901464, a metabolite from the bacterium *Pseudomonas sp.* No. 2663 [4], [16]. SSA potently inhibits splicing both *in vivo* and *in vitro*, and also exhibits anti-cancer activity [4], [16], [17]. SSA binds to the SF3b complex, a sub-component of U2 snRNP [4], [18], thereby preventing a stable interaction between pre-mRNA and SF3b1, the largest subunit of SF3b, and reducing the fidelity of branch-point recognition [19]. Another method for inhibiting splicing employs antisense morpholino oligos (AMOs). AMOs block RNA–RNA interactions between snRNA and pre-mRNA, resulting in functional knockdown of snRNPs [14], [15], [20].

In this study, we found that U2 snRNP inhibition by SSA caused the accumulation of RNA polymerase II (Pol II) near the 5′ end of genes in a gene-specific manner, resulting in the expression of only the upstream regions of some genes. Expression of the 3′ ends of genes was restored after the removal of SSA from the culture medium. These results suggest that U2 snRNP is important for expression of full-length genes. We propose that this mechanism serves as a novel quality-control and checkpoint mechanism that prevents pre-mRNA accumulation and aberrant protein production when splicing is defective.

## Materials and Methods

### Cell lines, antibodies, reagents and transfection

HeLa S3 cells were cultured in Dulbecco's modified Eagle's medium containing 10% heat-inactivated fetal bovine serum (Sigma). Anti-RRP4 (ab156698) antibody was purchased from Abcam. Anti-α-tubulin (B-5-1-2) antibody was purchased from Sigma. Anti–Pol II (N20) antibody was purchased from Santa Cruz Biotechnologies. HRP-conjugated anti-mouse IgG and anti-rabbit IgG were purchased from GE Healthcare. SSA was prepared as described previously [4]. Control siRNA (siGENOME Non-Targeting siRNA Pool #1) and RRP4 siRNA were purchased from Thermo Scientific. siRNA transfection was performed using Lipofectamine RNAiMAX Reagent (Life Technologies). The sequences of U1, U2, and control AMOs were 5′-GGTATCTCCCCTGCCAGGTAAGTAT-3′, 5′-TGATAAGAACAGATACTACACTTGA-3′ and 5′-CCTCTTACCTCAGTTACAATTTATA-3′, respectively; AMOs were purchased from Gene Tools, LLC. AMO transfection was performed using the Neon Transfection System (Life Technologies). HeLa cells (5×10^5^ cells) were trypsinized, washed twice with PBS, and resuspended in 100 µl of the resuspension buffer. After mixing cells with AMOs, electroporation was performed with the following parameters: 1300 V, 10 msec, 3 pulses.

### RNA preparation, 3′-RACE, and quantitative RT-PCR

Purification of sample RNA was performed using the Click-iT Nascent RNA Capture Kit (Life Technologies). Cells were treated with 200 µM of 5-ethynyl-uridine for 1 hour, and total RNA was extracted from cultured cells using TRIzol (Life Technologies). Labeled RNA was biotinylated by the click reaction, and biotinylated RNA was purified using streptavidin beads. For quantitative RT-PCR, cDNA was synthesized using the SuperScript VILO cDNA Synthesis Kit (Life Technologies). Quantitative RT-PCR was performed using 2.5 µl of cDNA, and relative quantification analyses were performed with an MX3000P system (Agilent) using SYBR Green dye chemistry. The amount of newly synthesized 5S rRNA was measured as an internal control. For 3′-RACE, cDNA was synthesized using the same labeled RNA and PrimeScript Reverse Transcriptase (TAKARA) using oligo dT18-XbaKpnBam primer. 3′ RACE was carried out using the first and second (nested) forward primers and the XbaKpnBam reverse primer. All primers are listed in Table S3.

### ChIP assay

For crosslinking, HeLa cells were treated with 1% formaldehyde for 10 min at room temperature. Crosslinking was stopped by the addition of glycine (final concentration: 125 mM) and cells were incubated for 5 minutes at room temperature. Cells were washed with cold PBS twice and collected by centrifugation (2500 rpm, 10 min, 4°C). After centrifugation, sheared chromatin DNA was prepared using the Bioruptor (Cosmo Bio). Approximately 8 µg of sheared chromatin was incubated overnight at 4°C with 2 µg of anti-Pol II antibody (N20). Thirty microliters of a slurry of Dynabeads Protein G (Life Technologies) was rotated overnight at 4°C in PBS containing 1 mg/ml BSA and 0.2 mg/ml of salmon sperm DNA. Antibody-bound chromatin was incubated with blocked Dynabeads Protein G for 2.5 h at 4°C. Immunoprecipitants were washed three times with a low-salt wash buffer (20 mM Tris-HCl [pH 8.0], 2 mM EDTA [pH 8.0], 150 mM NaCl, 1% Triton X-100 and 0.1% SDS), once with high-salt wash buffer (20 mM Tris-HCl [pH 8.0], 2 mM EDTA [pH 8.0], 500 mM NaCl, 1% Triton X-100, and 0.1% SDS) and then once with LiCl wash buffer (10 mM Tris-HCl [pH 8.0], 1 mM EDTA [pH 8.0], 250 mM LiCl, 1% NP-40, and 1% Na-deoxycholate). Beads were incubated with elution buffer (1% SDS, 0.1 M Na-bicarbonate, and 200 mM NaCl) at 65°C overnight, followed by a 1-hour incubation with 40 ng/µl of RNase A at 37°C and a 2-hour incubation with 40 ng/µl of proteinase K at 45°C. The DNA fragments were purified using the QIAquick PCR Purification Kit (Qiagen) and analyzed by quantitative PCR. Input DNA was analyzed simultaneously for normalization. All primers are listed in Table S3.

### Exon-array target preparation, hybridization, and analysis

HeLa cells (1×10^6^ cells) were treated with or without 100 ng/ml of SSA for 4 hours, and RNA was labeled during transcription with 5-ethynyl-uridine (200 µM) between 3 and 4 hours after the addition of SSA. Total RNA was extracted from cultured cells using TRIzol (Life Technologies); purified RNA was biotinylated by the click reaction, and then purified using streptavidin beads. Labeled cDNA targets were prepared using the Ambion WT Expression Kit (Life Technologies) and GeneChip WT Terminal Labeling Kit (Affymetrix). End-labeled cDNA targets were applied to GeneChip Human Exon 1.0 ST arrays (Affymetrix). Hybridization and scanning were performed using the GeneChip Hybridization, Wash, and Stain Kit (Affymetrix) and the GeneChip Scanner 3000 7G system (Affymetrix) to produce.CEL files. Probeset intensities were calculated from these.CEL files using Partek Genomic Suite 6.5 with default settings at the probeset level, using the core probe sets as defined by Affymetrix. Probesets with low expression levels (mean <3) and high statistical dispersion (standard deviation greater than 10% of the mean) were excluded. The gene lengths and exon numbers of genes were analyzed with Archive EnsEMBL (http://apr2011.archive.ensembl.org/index.html). P-values were calculated using a script written in R (http://www.r-project.org/). The exon array data from this publication have been submitted to the GEO database (http://www.ncbi.nlm.nih.gov/geo/) and assigned the identifier GSE45379.

### Western blotting

HeLa cells were lysed on plates in 1× SDS-PAGE sample buffer. Proteins were separated by SDS-PAGE. After electrophoresis, proteins were transferred onto a PVDF membrane by electroblotting. After the incubation of these membranes with primary and secondary antibodies, immune complexes were detected using the NOVEX ECL Chemiluminescent Substrate Reagent Kit (Life Technologies) on an ImageQuant LAS 4000mini (GE Healthcare). Band intensities were quantified using the ImageQuant TL software (GE Healthcare).

## Results

### SSA treatment causes 3′-end down-regulation

**Figure 1:**
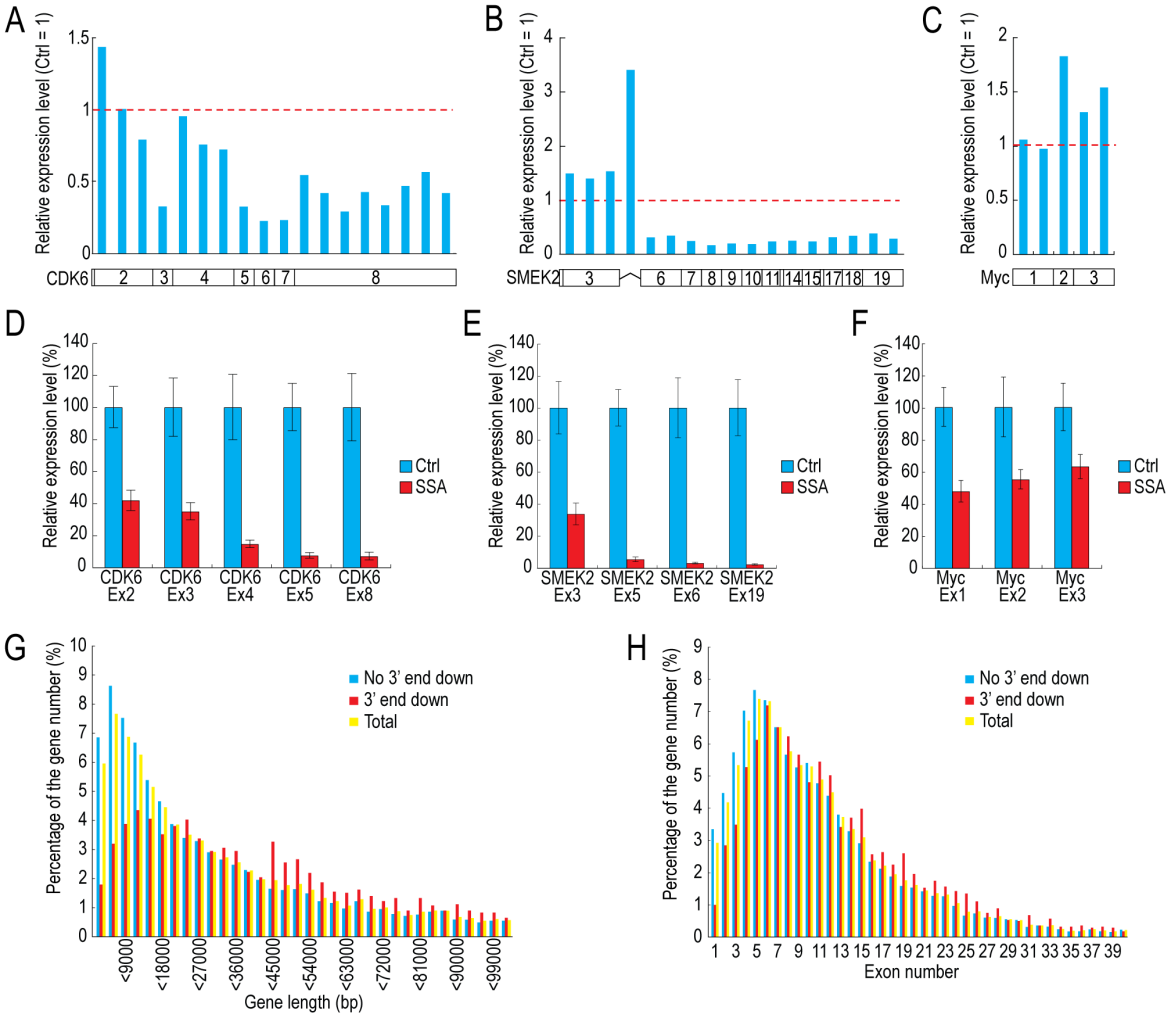
Selected genes exhibiting 3′-end down-regulation in SSA-treated cells. (A, B, C) HeLa cells were treated with SSA (100 ng/ml), and RNAs were labeled during transcription with EU between 3 and 4 hours after the addition of SSA. Labeled RNAs were analyzed using human exon arrays. Fold changes in the signal intensities of SSA-treated cells relative to those in methanol-treated cells (controls) are shown above the corresponding regions in the gene structures. (D, E, F) HeLa cells were treated with SSA (100 ng/ml), and RNAs were labeled during transcription with EU between 3 and 4 hours after the addition of SSA. Labeled RNAs were analyzed by quantitative RT-PCR in order to measure the relative expression levels of selected exons of three genes (methanol-treated cells [Ctrl]  = 100%). Error bars indicate s.d. (n = 3). (G) The size distributions of genes with (magenta) or without (cyan) 3′-end down-regulation, and of all genes (yellow). (H) The exon-number distributions of genes with (magenta) or without (cyan) 3′-end down-regulation, and of all genes (yellow).

To investigate the effect of U2 snRNP inhibition by SSA treatment on the transcriptome, we treated HeLa cells with SSA and monitored the resultant changes in the transcriptome of SSA-treated cells. We postulated that brief SSA treatment (i.e., for a few hours) would be insufficient to cause changes in the levels of stable mRNAs with long half-lives, whereas long SSA treatment would be likely to cause secondary effects. To resolve this technical challenge and sensitively observe the effects of splicing inhibition on the transcriptome, we analyzed only RNAs that were synthesized after splicing inhibition, and disregarded the mRNAs already present prior to SSA treatment. To this end, HeLa cells were treated with SSA for 4 hours; RNAs were labeled during transcription with 5-ethynyl-uridine (EU), a uridine derivative, between 3 and 4 hours after the addition of SSA, and then the labeled RNAs were purified and analyzed. To obtain a global picture of the changes occurring in the transcriptome of SSA-treated cells, we analyzed RNAs from three independent experiments using Affymetrix human exon arrays. Out of ∼230,000 probe sets on the array, more than 170,000 probe sets with small standard deviations, corresponding to ∼15,800 genes, were included in the analysis. To provide illustrative examples, the expression level of each probe set of the *CDK6*, *SMEK2* and *C-MYC* genes in SSA-treated cells, relative to the corresponding level in control cells, is shown in Figure 1A–C. The expression levels of exons of *CDK6* and *SMEK2* gradually decreased from the 5′ end to the 3′ end, whereas the exons of *C-MYC* did not exhibit this property (Fig. 1A–C). This phenomenon was also confirmed by RT-qPCR (Fig. 1D–F). SSA treatment has been reported to cause alternative splicing [19]; however, we observed no exon skipping in SSA-treated cells, at least in these three genes.

We defined as 3′-end down-regulated those genes for which the ratio of the relative expression of the most downstream probe set to that of the most upstream probe set was less than 0.5. Out of the ∼15,800 genes analyzed, 2,799 (17.8%) were 3′-end down-regulated (Fig. 1, and S1, Table S1 and S2). To identify common features in 3′-end down-regulated genes, we first examined the size distribution of this gene set. Notably, 3′-end down-regulation occurred less frequently among relatively short genes (<15,000 bp) than among long genes (≥15,000 bp) (Fig. 1G). In addition, the average size of 3′-end down-regulated genes was significantly greater than that of genes that were not 3′-end down-regulated (p = 2.494×10^−7^). Next, we examined the exon number of 3′-end down-regulated genes. Genes with small numbers of exons (from one to five exons) were not frequently 3′-end down-regulated (Fig. 1H). The average number of exons of 3′-end down-regulated genes was significantly greater than that of genes not 3′-end down-regulated (p = 2.2×10^−16^). Because genes with fewer introns are likely to be relatively short, these data suggest that gene length is the critical factor in determining 3′-end down-regulation (See Discussion).

### U2 snRNP inhibition correlates with 3′-end down-regulation

**Figure 2:**
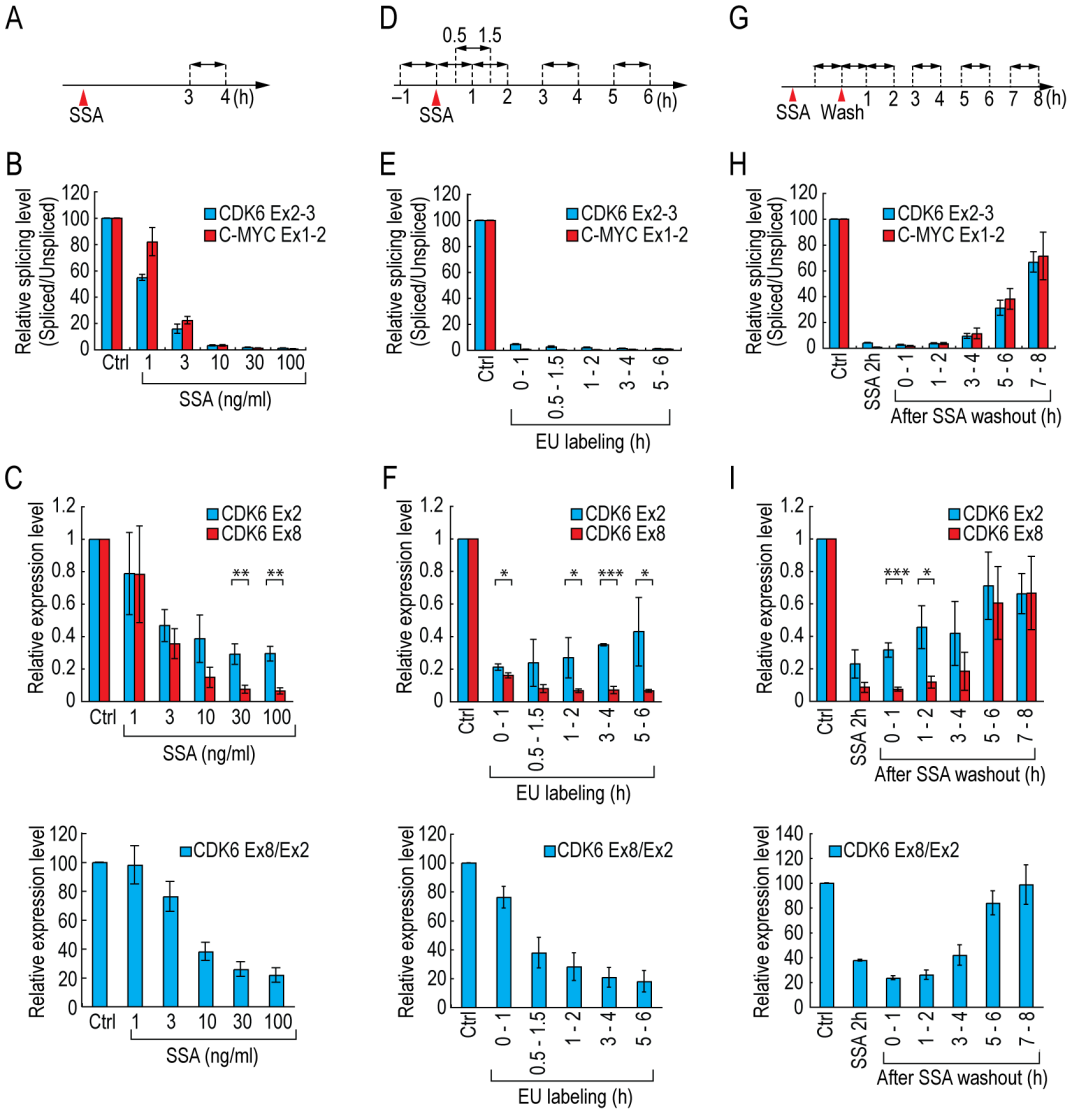
SSA treatment causes splicing inhibition and 3′-end down-regulation. (A, B, C) HeLa cells were treated with the indicated concentrations of SSA for 4 hours, and RNAs were labeled during transcription with EU between 3 and 4 hours after the addition of SSA (A). Labeled RNAs were analyzed by quantitative RT-PCR to measure the amounts of spliced and unspliced mRNA. Relative splicing activity was defined as the ratio of spliced to unspliced mRNA (methanol-treated cells [Ctrl]  = 100%) (B). The levels of the 5′ and 3′ ends of *CDK6* (Exon 2 and Exon 8, respectively) were measured by quantitative RT-PCR (C, upper panel), and the ratio between the 3′ and 5′ levels was calculated (methanol-treated cells [Ctrl]  = 100%) (C, lower panel). (D, E, F) HeLa cells were treated with 100 ng/ml of SSA, and RNAs were labeled during transcription with EU for 1 hour as indicated (D). Labeled RNAs were analyzed as in Figure 2B and 2C (methanol-treated cells [Ctrl]  = 100%) (E, F). (G, H, I) HeLa cells were treated with 100 ng/ml of SSA for 2 hours, and then washed with fresh medium. Cells were cultivated in fresh medium, and RNAs were labeled during transcription with EU for 1 hour as indicated (G). Labeled RNAs were analyzed as in Figure 2B and 2C (methanol-treated cells [Ctrl]  = 100%) (H, I). Error bars indicate s.d. (n = 3). Statistical significance was investigated by the t-test (*: p<0.05; **: p<0.01; ***: p<0.001).

In order to determine whether U2 snRNP inhibition by SSA treatment caused 3′-end down-regulation, we measured splicing levels and relative expression levels of the 3′ and 5′ ends of selected genes in cells treated with different amounts of SSA, because splicing level is a measure of U2 snRNP activity. We treated HeLa cells with SSA, and labeled RNAs during transcription with EU between 3 and 4 hours after the addition of SSA (Fig. 2A). Consistent with previous reports, mRNA splicing of both *CDK6*, a 3′-end down-regulated gene, and *C-MYC*, which is not 3′-end down-regulated, was inhibited by SSA treatment in a dose-dependent manner (Fig. 2B) [4]. Next, we examined the expression levels of the 5′ and 3′ ends of *CDK6*. The expression level of the 5′ end (*CDK6* Ex2) declined gradually as the concentration of SSA increased; however, the level of the 3′ end (*CDK6* Ex8) decreased more sharply than that of the 5′ end (Fig. 2C). Consequently, the ratio between the 3′ and 5′ levels was decreased by SSA treatment in a dose-dependent manner (Fig. 2C). The pattern of splicing activity was very similar to that of the relative level of the 3′ end (Fig. 2B and 2C). In addition to *CDK6*, *SMEK2* and another 3′-end down-regulated gene, *VEGFA*, exhibited similar patterns (Fig. S1A and S2). These results suggest that splicing level and U2 snRNP activity negatively correlate with 3′-end down-regulation.

Next, we investigated the time-dependency of U2 snRNP inhibition and 3′-end down-regulation caused by SSA treatment. To this end, we treated HeLa cells with SSA, and labeled transcripts during transcription with EU for 1 hour starting at different times after the onset of SSA treatment (Fig. 2D). Almost no spliced *CDK6* or *C-MYC* mRNA was observed during the first hour of SSA treatment (Fig. 2E), suggesting that U2 snRNP and splicing reaction were completely inhibited immediately after addition of SSA. On the other hand, 3′-end down-regulation was observed slightly later than splicing inhibition (Fig. 2E, 2F and S3), consistent with the idea that inhibition of U2 snRNP and splicing occurs mechanistically upstream of 3′-end down-regulation.

SSA does not bind to its target protein covalently [4]; therefore, U2 snRNP function and splicing activity should eventually recover after removal of SSA from the culture media. To determine whether levels of 3′ ends would recover concomitantly with the recovery of splicing level, we treated HeLa cells with SSA for 2 hours, washed out the SSA from the culture media, and then measured splicing level and relative levels of 3′ ends (Fig. 2G). As we expected, splicing activity recovered to control levels 7–8 hours after the SSA washout (Fig. 2H). Expression of the 3′ ends of the genes also recovered almost simultaneously with splicing level (Fig. 2H, 2I and S4). These results suggest that inhibition of U2 snRNP and splicing by SSA is the cause of gene-specific 3′-end down-regulation.

Therefore, we next investigated in detail the relationship between U2 snRNP activity and 3′-end down-regulation. Because *CDK6* and *SMEK2* exhibited 3′-end down-regulation but *C-MYC* did not, we speculated that *CDK6* and *SMEK2* have introns that are sensitive to U2 snRNP inhibition by SSA, and the splicing level after SSA treatment might differ among these three genes. Alternatively, splicing level might decrease from the 5′ to 3′ ends of genes, and this could contribute to 3′-end down-regulation. To test these possibilities, we measured the relative splicing levels of *CDK6*, *SMEK2* and *C-MYC*, and observed significant splicing inhibition in all introns we tested. The strongest splicing inhibition was observed on *C-MYC*, which is not 3′-end down-regulated (Fig. S5). We also found that splicing level did not decrease from the 5′ to the 3′ end (Fig. S5). These results suggest that the splicing status of a gene does not directly influence its transcription elongation.

### The short transcripts in SSA-treated cells are not prematurely polyadenylated

**Figure 3:**
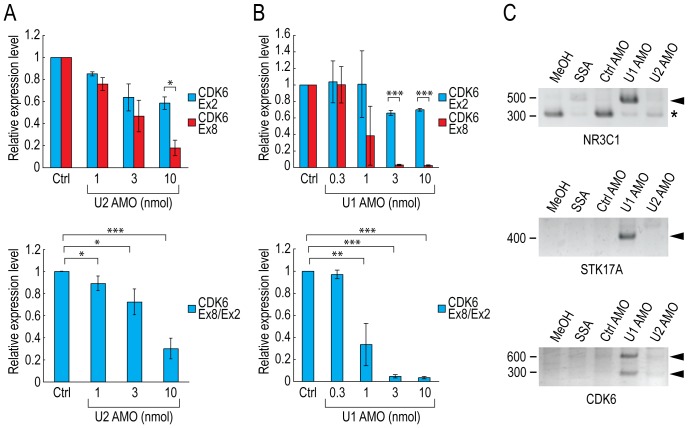
PCPA does not cause 3′-end down-regulation in SSA- and U2 AMO–treated cells. (A, B) HeLa cells were transfected with varying concentrations of U2 AMO (A) or U1 AMO (B) as indicated, and then cultured for 6 hours after transfection. RNAs were labeled during transcription with EU between 5 and 6 hours after transfection, and labeled RNAs were analyzed by quantitative RT-PCR. The levels of the 5′ ends of CDK6 gene (CDK6 Exon 2) and the 3′ ends of CDK6 gene (CDK6 Exon 8) were measured (upper panels), and the ratio between the 3′ and 5′ levels was calculated (methanol-treated cells [Ctrl]  = 100%) (lower panels). Error bars indicate s.d. (n = 3). Statistical significance was investigated by the t-test (*: p<0.05; **: p<0.01; ***: p<0.001). (C) HeLa cells were treated with MeOH (vehicle) or SSA (100 ng/ml), or transfected with Control (3 nmol), U1 (3 nmol), or U2 AMO (10 nmol), and then cultivated for 6 hours. RNAs were labeled during transcription with EU between 5 and 6 hours after the initiation of treatment, and labeled RNAs were analyzed by 3′-RACE. Band sizes (bp) are indicated to the left of the gels. Bands representing RNA that has undergone PCPA (Arrowheads) and spliced mRNA (*) are indicated to the right of the gels.

The SF3b complex is the primary target of SSA; however, it is also possible that SSA causes 3′-end down-regulation by binding to another target protein. To rule out this possibility, we treated HeLa cells with antisense morpholino oligos (AMOs) against the U2 snRNA [14], [15], and then measured the relative levels of the 3′ ends of selected genes. If 3′-end down-regulation is caused by direct inhibition of U2 snRNP, rather than by a side effect of SSA, we would predict the same effect when U2 snRNP is inhibited by U2 AMO. At first, we confirmed functional knockdown of U2 snRNP by U2 AMO by measuring the splicing level (Fig. S6). In U2 AMO–treated cells, the levels of the 5′ ends of the selected genes gradually declined, and the levels of 3′ ends decreased more sharply, as the concentration of U2 AMO increased (Fig 3A and S7). Consequently, the ratios between the 3′ and 5′ levels were statistically significantly decreased by U2 AMO in a dose-dependent manner (Fig 3A and S7), which is consistent with our observations in SSA-treated cells (Fig. 2C, 3A, S2 and S7). This finding suggests that 3′-end down-regulation is the consequence of U2 snRNP inhibition, rather than a side effect of SSA.

The levels of 5′ ends of selected genes decreased in SSA- and U2 AMO–treated cells; however, in U1 snRNP inhibited-cells by U1 AMO, the levels of 5′ ends did not decrease, and in some cases even increased (Fig. 2C, 3A, 3B, S2 and S7). Functional knockdown of the U1 snRNP results in premature termination of transcription, caused by premature cleavage and polyadenylation (PCPA) [14], [20]. These results indicate that those genes were cleaved and polyadenylated prematurely in U1 AMO–treated cells, and that the 5′ ends of the RNAs were consequently stabilized, because the poly(A) tail prevented degradation of prematurely terminated RNAs. However, the levels of the same regions were reduced in SSA- and U2 AMO–treated cells, suggesting that these regions did not have poly(A) tails and were not stable. We conclude that PCPA occurred in U1 AMO–treated cells, as previously reported [14], [20]; however, PCPA did not seem to explain the 3′-end down-regulation observed in SSA- and U2 AMO–treated cells, at least for the genes we tested.

Next, to show directly that PCPA does not cause the 3′-end down-regulation in SSA- and U2 AMO–treated cells, we performed 3′ rapid amplification of cDNA ends (3′-RACE) [14], [20]. As we predicted, we observed PCPA in the *NR3C1* and *STK17A* genes only in U1 AMO–treated cells, but observed very little if any PCPA in U2 AMO– or SSA-treated cells (Fig. 3C), as previously reported [14]. In addition to these two genes, we searched for PCPA sites in 3′-end down-regulated genes and identified two PCPA sites in *CDK6*. Two bands representing RNAs that had undergone PCPA were observed only in U1 AMO–treated cells (Fig. 3C), and both of these RNAs contained the polyadenylation signal AATAAA ∼20 nt upstream of the poly(A) tail (Fig. S8). These results suggest that the 3′-end down-regulation observed in SSA- and U2 AMO–treated cells was not caused by PCPA, although it is possible that a small portion of genes are subjected to PCPA.

### 3′-end down-regulation occurs in RRP4 knockdown cells

**Figure 4:**
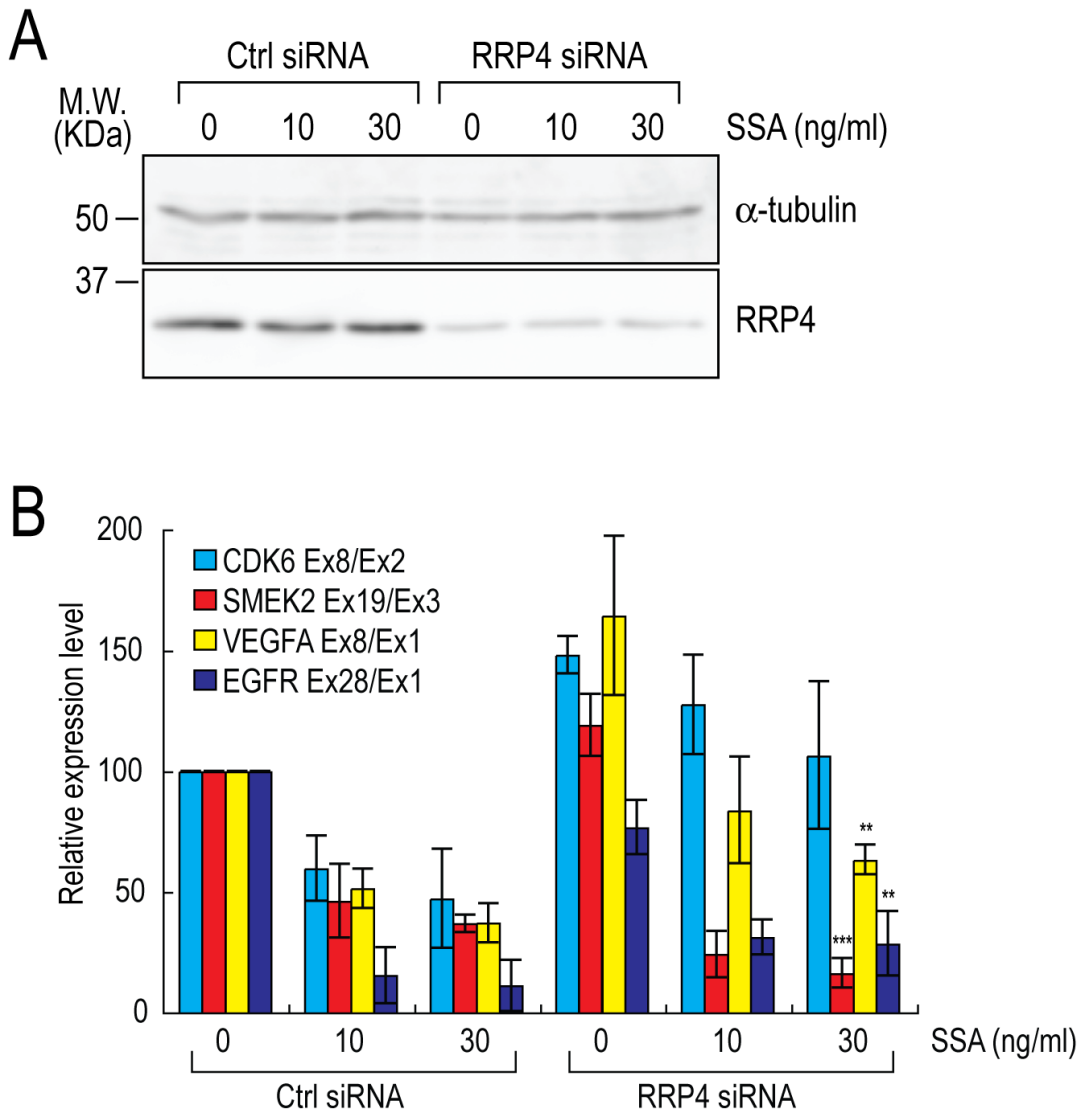
3′-end down-regulation by SSA treatment is observed in exosome deficient cells. (A, B) HeLa cells were transfected with Control or *RRP4* siRNA (20 nM), and then cultured for 48 hours after transfection. Cells were then treated with the indicated concentration of SSA for 4 hours, and RNAs were labeled during transcription with EU between 3 and 4 hours after the addition of SSA. (A) Half of the transfected cells were analyzed by Western blotting to measure the level of RRP4 protein. Molecular weights are indicated to the left of the gels. (B) RNA samples were purified from the other half of the cells, and labeled RNAs were analyzed by quantitative RT-PCR. The ratios between the 3′ and 5′ ends of *CDK6*, *SMEK2*, *VEGFA* and *EGFR* were calculated (methanol-treated and control siRNA-transfected cells  = 100%). Error bars indicate s.d. (n = 3). Statistical significance (RRP4 siRNA, 0 ng/ml SSA vs. RRP4 siRNA, 30 ng/ml SSA) was investigated by the t-test (**: p<0.01; ***: p<0.001).

The nuclear exosome complex degrades pre-mRNAs accumulated as a result of splicing defects; degradation proceeds from the 3′ end to the 5′ end [12]. Therefore, it is possible that 3′-end down-regulation in SSA-treated cells is a consequence of the partial degradation of the 3′ ends of pre-mRNAs. To test this possibility, we transfected cells with siRNAs targeting *RRP4* mRNA, which encodes a component of the exosome complex [21]. After treatment with siRNAs against *RRP4*, the RRP4 protein level significantly decreased (Fig. 4A). We observed a significant 3′-end down-regulation of *SMEK2* and *EGFR*, another 3′-end down-regulated gene, upon SSA treatment of RRP4-knockdown cells (Fig. 4B and S1B), suggesting that 3′-end down-regulation occurs even in the exosome deficient cells. *CDK6* and *VEGFA* also showed 3′-end down-regulation by SSA treatment in RRP4-knockdown cells, however the effects were weaker than *SMEK2* and *EGFR*. Since mRNA turnover in the cytoplasm and degradation of some portion of pre-mRNAs prior to splicing are carried out by the exosome complex [22], [23], *CDK6* and *VEGFA* mRNAs might be good targets of the exosome complex and might be degraded from their 3′ end. Actually, the ratios between the 3′ and 5′ levels were increased in *CDK6* and *VEGFA* in RRP4-knockdown cells (Fig. 4B, RRP4 siRNA, 0 ng/ml SSA). Presumably, 3′-end down regulation of *CDK6* and *VEGFA* following SSA treatment was counteracted by 3′-end up-regulation caused by RRP4 knockdown; therefore, 3′-end down regulation of *CDK6* and *VEGFA* seemed to be weaker than *SMEK2* and *EGFR*. These results suggest that 3′-end down-regulation occurs even in the exosome deficient cells, although we cannot rule out the possibility that degradation by the exosome partially contributes 3′-end down-regulation.

### Pol II pauses near the 5′ end of 3′-end down-regulated genes in SSA-treated cells

**Figure 5:**
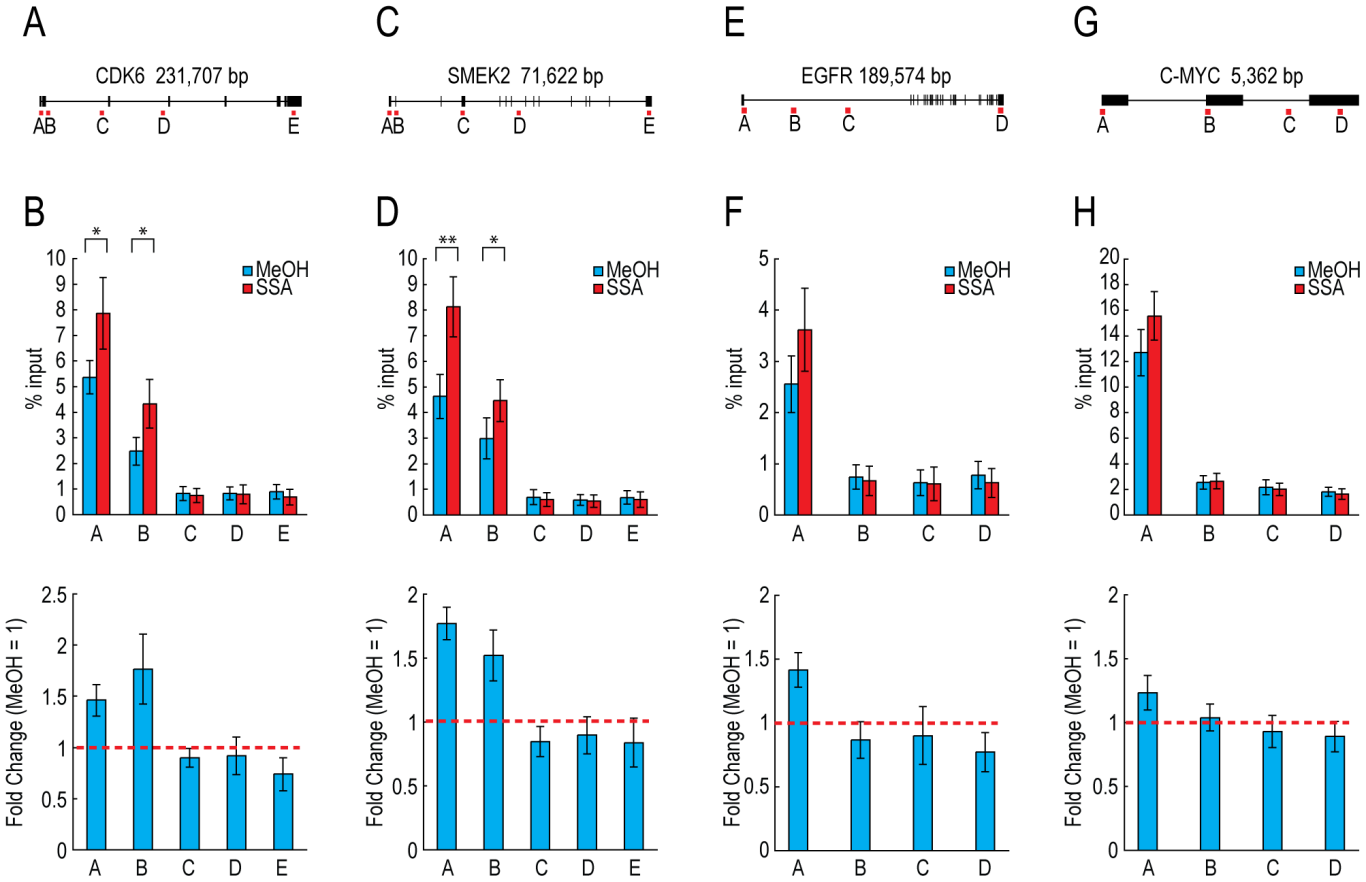
Pol II accumulates near the 5′ end of genes. (A, C, E, G) The locations of primers used in this assay are shown under the gene structures. (B, D, F, H) HeLa cells were treated with 30 ng/ml of SSA for 2 hours, and the distributions of Pol II were analyzed by chromatin immunoprecipitation (ChIP). The amount of immunoprecipitated DNA fragments was measured by quantitative PCR (upper panels). The ratios between the percent input of SSA-treated cells (SSA) and methanol-treated cells (MeOH) were calculated (MeOH  = 1) (lower panels). Error bars indicate s.d. (n = 4). Statistical significance was investigated by the t-test (*: p<0.05; **: p<0.01).

Because PCPA and degradation by the nuclear exosome did not explain completely the 3′-end down-regulation resulting from treatment with SSA and U2 AMO, we speculated that transcription elongation of 3′-end down-regulated genes is defective in SSA-treated cells. To investigate the distribution of Pol II after SSA treatment, we performed chromatin immunoprecipitation (ChIP) assays on selected genes after SSA treatment, and found that Pol II accumulated near the 5′ end of 3′-end down-regulated genes, *CDK6*, *SMEK2* and *EGFR*, although Pol II accumulation on *EGFR* gene was less significant than *CDK6* and *SMEK2* (Fig. 5). By contrast, almost no significant change was observed on *C-MYC* (Fig. 5). These results indicate that U2 snRNP inhibition by SSA treatment leads to 3′-end down-regulation, presumably caused by gene-specific Pol II pausing near the 5′ end of gene.

## Discussion

Quality-control mechanisms, such as NMD or tethering and degradation of pre-mRNA in the nucleus, prevent translation from precursor or aberrant mRNAs [7], [8], [9], [10], [11], [12]. The results of this study suggest that a novel quality-control and checkpoint mechanism related to splicing activity also helps to protect the integrity of the transcriptome. Inhibition of U2 snRNP by SSA and U2 AMO resulted in the gene specific pausing of Pol II near the 5′ end and down-regulation of the 3′ end. This might function to prevent the pre-mRNA accumulation and the translation from such pre-mRNAs. These results are consistent with those of recent reports [24], [25]. They did show that the distribution of Pol II was affected by SSA treatment in a gene-specific manner. These findings suggest that U2 snRNP activity is required for efficient transcription elongation. Interestingly, all three genes we tested, *CDK6, SMEK2* and *C-MYC*, showed significant splicing inhibition (Fig. 2 and S5), however, only *CDK6* and *SMEK2* showed the 3′ end down-regulation (Fig. 1). These results suggest that the splicing status of a gene does not directly influence its transcription elongation, but that total U2 snRNP activity in cells is important (see below).

The gene-specific pausing of Pol II and 3′-end down-regulation in SSA-treated cells reminded us of a recent report showing that the splicing factor SC35 is required for efficient transcription elongation of some genes [26], [27], consistent with our results. More recently, it was reported that Prp5 protein, which is required for pre-spliceosome formation is important for transcription elongation by J. Beggs' group [28]. These reports strongly suggest that some splicing factors and spliceosome formation are required for efficient transcription elongation. However, contrary to our findings, it has also been reported that splicing inhibition by SSA treatment does not affect transcription elongation [29]. This discrepancy can be explained as follows: Brody et al. [29] used a short reporter gene (∼5 kb) in their assay, whereas this study revealed that short genes (<15 kb) do not often exhibit transcription-elongation defects. Therefore, gene length is a determinant of 3′-end down-regulation, although it is not the only factor (see below).

Why are relatively short genes resistant to 3′-end down-regulation? This observation might be explained by the presence on the DNA template of roadblocks that prevent efficient transcription. For example, due to the strong interaction between DNA and histones, nucleosomes can be roadblocks [30], [31], [32]. In addition, DNA-binding proteins such as MAZ and Vezf also interfere with Pol II elongation, [33], [34], [35]. When elongating Pol II encounters these roadblocks, it pauses or arrests [36], [37]. Long genes have a higher probability of containing such roadblocks than short genes; we speculate that long genes that did not exhibit 3′-end down-regulation lack such barriers (Fig. 1G). Under normal conditions, Pol II overcomes roadblocks with help from other transcriptional activators [36], [37], [38], [39]. Since inhibition of U2 snRNP causes gene-specific pausing of Pol II and 3′-end down-regulation, U2 snRNP might be such a transcriptional activator, or might recruit such activators.

As mentioned above, decrease of the ratio between the 3′ and 5′ end of *VEGFA* after U2 inhibition was statistically significant, however it was less sharp than that of *CDK6* and *SMEK2* (Fig. 2, 3, S2, S3 and S7). Since recruitment of Pol II to *VEGFA* promoter and transcription initiation of *VEGFA* gene are impaired in U2 inhibited cells by SSA [40], we assume that this impairment makes it hard to see the difference between 3′ end level and the 5′ end level, and consequently affects the ratio of 3′ end and 5′ end of *VEGFA* gene.

What is the physiological significance of this mechanism in cells? Splicing abnormalities risk accumulation of pre-mRNAs, which might result in the translation of pre-mRNAs into truncated nonfunctional (or even toxic) proteins. Cells have several quality-control mechanisms to prevent the translation of pre-mRNAs [7], [8], [9], [10], [11], [12]. Arrest of elongation at a splicing abnormality, a phenomenon revealed in this study, represents a novel quality-control system. By preventing production of nonfunctional mRNAs, this system saves energy; by contrast, conventional quality-control systems are energetically unfavorable, because aberrant mRNAs are transcribed once and then either degraded or tethered in the nucleus.

This study identified a novel checkpoint and quality-control mechanism that ensures that only properly processed mRNAs will be translated, although the detailed molecular mechanism remains to be elucidated. We anticipate that identification and characterization of this mechanism will be of great interest, and will open the way to a better understanding of mRNA processing and quality-control and checkpoint mechanisms.

## Supporting Information

Figure S1 SSA treatment causes gene-specific 3′-end down-regulation. HeLa cells were treated with SSA (100 ng/ml) and RNAs were labeled during transcription with EU between 3 and 4 hours after the addition of SSA. Labeled RNAs were analyzed using human exon arrays. Fold changes in the signal intensities of SSA-treated cells, relative to control cells, are shown above the corresponding region of each gene.
(TIF)

Figure S2 SSA treatment causes gene-specific 3′-end down-regulation in a dose-dependent manner. HeLa cells were treated with the indicated concentrations of SSA for 4 hours, and RNAs were labeled during transcription with EU between 3 and 4 hours after the addition of SSA, as in Figure 2A. Labeled RNAs were analyzed by quantitative RT-PCR to measure the levels of the 5′ ends (SMEK2 Ex1 and VEGFA Ex1) and the 3′ ends (SMEK2 Ex19 and VEGFA Ex8) of these genes (upper panels). The ratio between the 3′ and 5′ levels was calculated for each gene (non-treated cells [Ctrl]  = 100%) (lower panels). Error bars indicate s.d. (n = 3). Statistical significance was investigated by the t-test (*: p<0.05; **: p<0.01; ***: p<0.001).
(TIF)

Figure S3 SSA treatment causes 3′-end down-regulation in a time-dependent manner. HeLa cells were treated with 100 ng/ml of SSA, and RNAs were labeled during transcription with EU for 1 hour, as in Figure 2D. Labeled RNAs were analyzed by quantitative RT-PCR to measure the levels of the 5′ ends (SMEK2 Ex3 and VEGFA Ex1) and 3′ ends (SMEK2 Ex19 and VEGFA Ex8) of these genes (upper panels). The ratio between the 3′ and 5′ levels was calculated for each gene (non-treated cells [Ctrl]  = 100%) (lower panels). Error bars indicate s.d. (n = 3). Statistical significance was investigated by the t-test (*: p<0.05; **: p<0.01; ***: p<0.001).
(TIF)

Figure S4 Expression of the 3′ ends of genes recovers after SSA washout. HeLa cells were treated with 100 ng/ml of SSA for 2 hours and washed twice with fresh medium. Cells were cultivated in fresh medium, and RNAs were labeled during transcription with EU for 1 hour, as in Figure 2G. Labeled RNAs were analyzed by quantitative RT-PCR to measure the levels of the 5′ ends (SMEK2 Ex1 and VEGFA Ex1) and 3′ ends (SMEK2 Ex19 and VEGFA Ex8) of these genes (upper panels). The ratio between the 3′ and 5′ levels was calculated for each gene (non-treated cells [Ctrl]  = 100%) (lower panels). Error bars indicate s.d. (n = 3). Statistical significance was investigated by the t-test (*: p<0.05; **: p<0.01; ***: p<0.001).
(TIF)

Figure S5 SSA treatment causes global splicing inhibition. HeLa cells were treated with 100 ng/ml of SSA for 4 hours, and RNAs were labeled during transcription with EU between 3 and 4 hours after the addition of SSA. Labeled RNAs were analyzed by quantitative RT-PCR to measure the amounts of spliced and unspliced mRNA. Relative splicing activity was defined as the ratio of spliced to unspliced mRNA (methanol-treated cells [Ctrl]  = 100%). Error bars indicate s.d. (n = 3).
(TIF)

Figure S6 AMO treatment causes splicing inhibition. HeLa cells were transfected with varying concentrations of U2 AMO (A) or U1 AMO (B) as indicated, and then cultured for 6 hours after transfection. RNAs were labeled during transcription with EU between 5 and 6 hours after transfection, and labeled RNAs were analyzed by quantitative RT-PCR to measure the amounts of spliced and unspliced mRNA. Relative splicing activity was defined as the ratio of spliced to unspliced mRNA (methanol-treated cells [Ctrl]  = 100%). Error bars indicate s.d. (n = 3).
(TIF)

Figure S7 AMO treatment causes splicing inhibition. HeLa cells were transfected with varying concentrations of U2 AMO (A, B) or U1 AMO (C, D) as indicated, and then cultured for 6 hours after transfection. RNAs were labeled during transcription with EU between 5 and 6 hours after transfection, and labeled RNAs were analyzed by quantitative RT-PCR to measure the levels of the 5′ ends (SMEK2 Ex3 and VEGFA Ex1) and 3′ ends (SMEK2 Ex19 and VEGFA Ex8) of these genes (upper panels). The ratio between the 3′ and 5′ levels was calculated for each gene (non-treated cells [Ctrl]  = 100%) (lower panels). Error bars indicate s.d. (n = 3). Statistical significance was investigated by the t-test (*: p<0.05; **: p<0.01; ***: p<0.001).
(TIF)

Figure S8 Sequencing of the 3′-RACE products. Sequences of the 3′-RACE products for the upper and lower bands of the CDK6 gene are shown along with the corresponding genomic sequences (in black). The poly(A) tails are shaded, and the putative PASs are indicated by red outlines.
(TIF)

Table S1 List of genes showing 3' end down regulation.
(XLSX)

Table S2 List of genes that did not show 3' end down regulation.
(XLSX)

Table S3 List of primers used in this study.
(DOCX)
