# Supplementary figures and images for: U2 snRNP Is Required for Expression of the 3′ End of Genes
Source: PLoS One. 2014 May 20;9(5):e98015. doi: 10.1371/journal.pone.0098015 (PMC4028248; doi:10.1371/journal.pone.0098015)

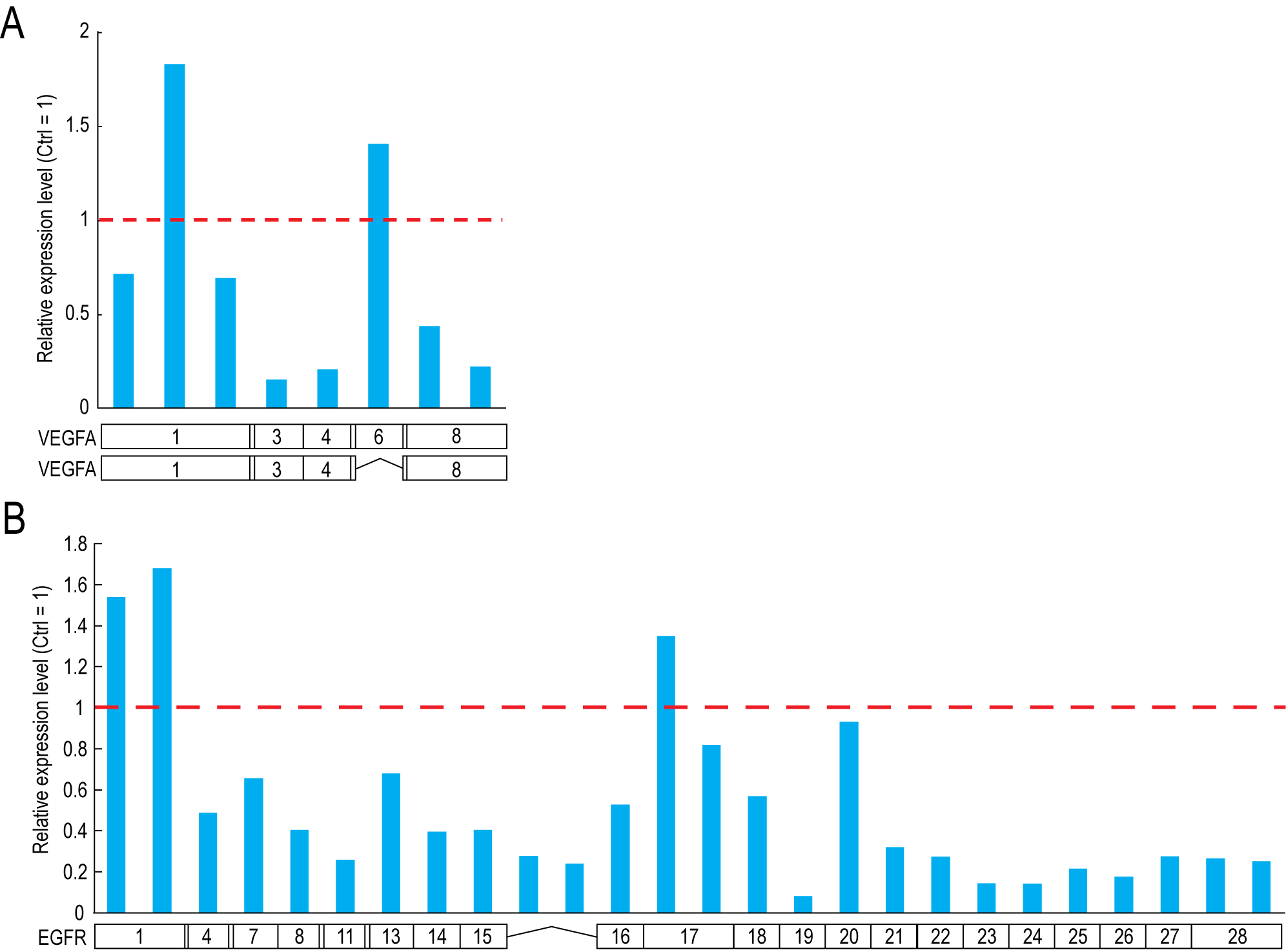

Supplement: Figure S1 — SSA treatment causes gene-specific 3′-end down-regulation. HeLa cells were treated with SSA (100 ng/ml) and RNAs were labeled during transcription with EU between 3 and 4 hours after the addition of SSA. Labeled RNAs were analyzed using human exon arrays. Fold changes in the signal intensities of SSA-treated cells, relative to control cells, are shown above the corresponding region of each gene. (TIF) [file pone.0098015.s001.tif]

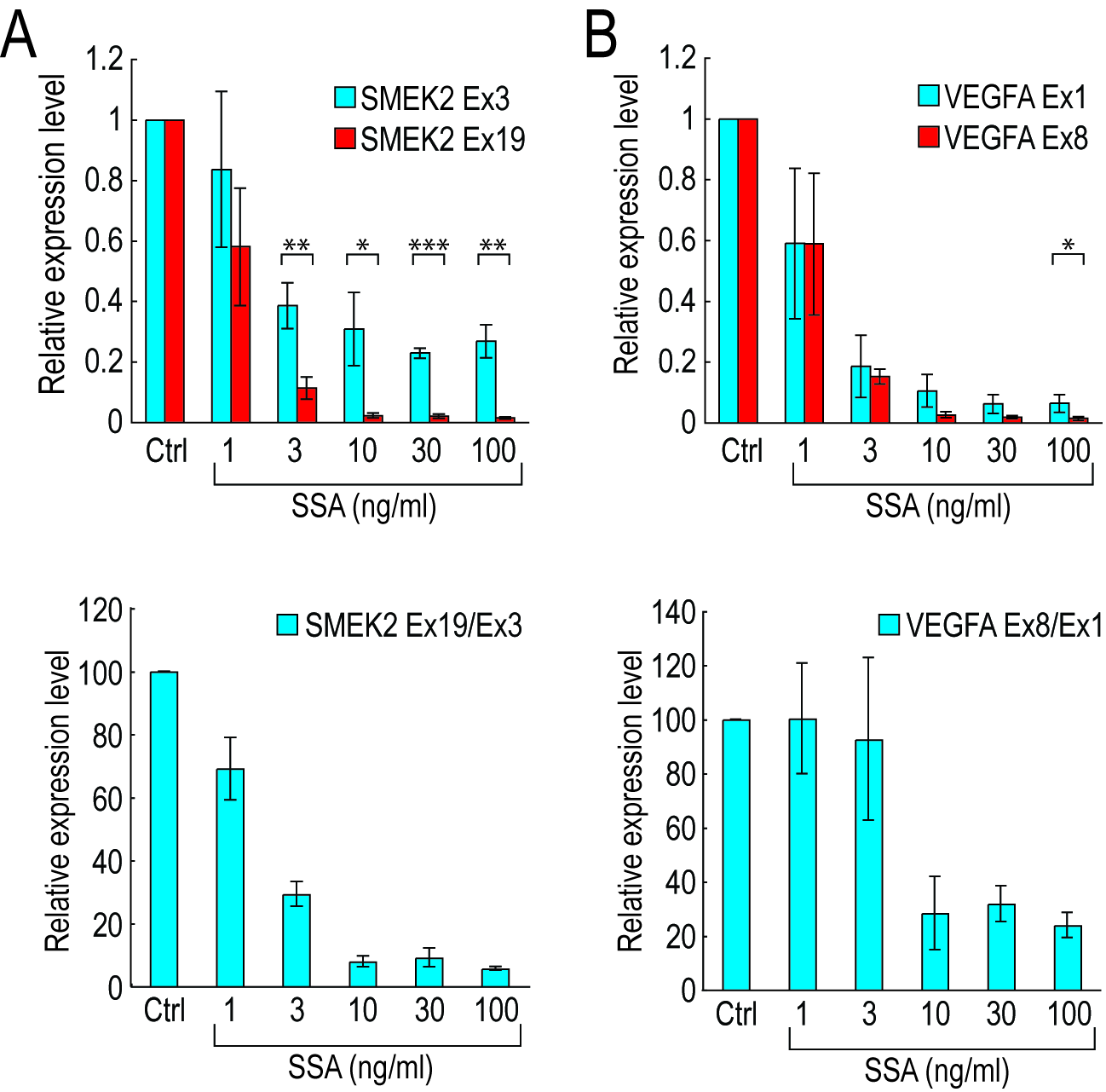

Supplement: Figure S2 — SSA treatment causes gene-specific 3′-end down-regulation in a dose-dependent manner. HeLa cells were treated with the indicated concentrations of SSA for 4 hours, and RNAs were labeled during transcription with EU between 3 and 4 hours after the addition of SSA, as in Figure 2A. Labeled RNAs were analyzed by quantitative RT-PCR to measure the levels of the 5′ ends (SMEK2 Ex1 and VEGFA Ex1) and the 3′ ends (SMEK2 Ex19 and VEGFA Ex8) of these genes (upper panels). The ratio between the 3′ and 5′ levels was calculated for each gene (non-treated cells [Ctrl] = 100%) (lower panels). Error bars indicate s.d. (n = 3). Statistical significance was investigated by the t-test (*: p<0.05; **: p<0.01; ***: p<0.001). (TIF) [file pone.0098015.s002.tif]

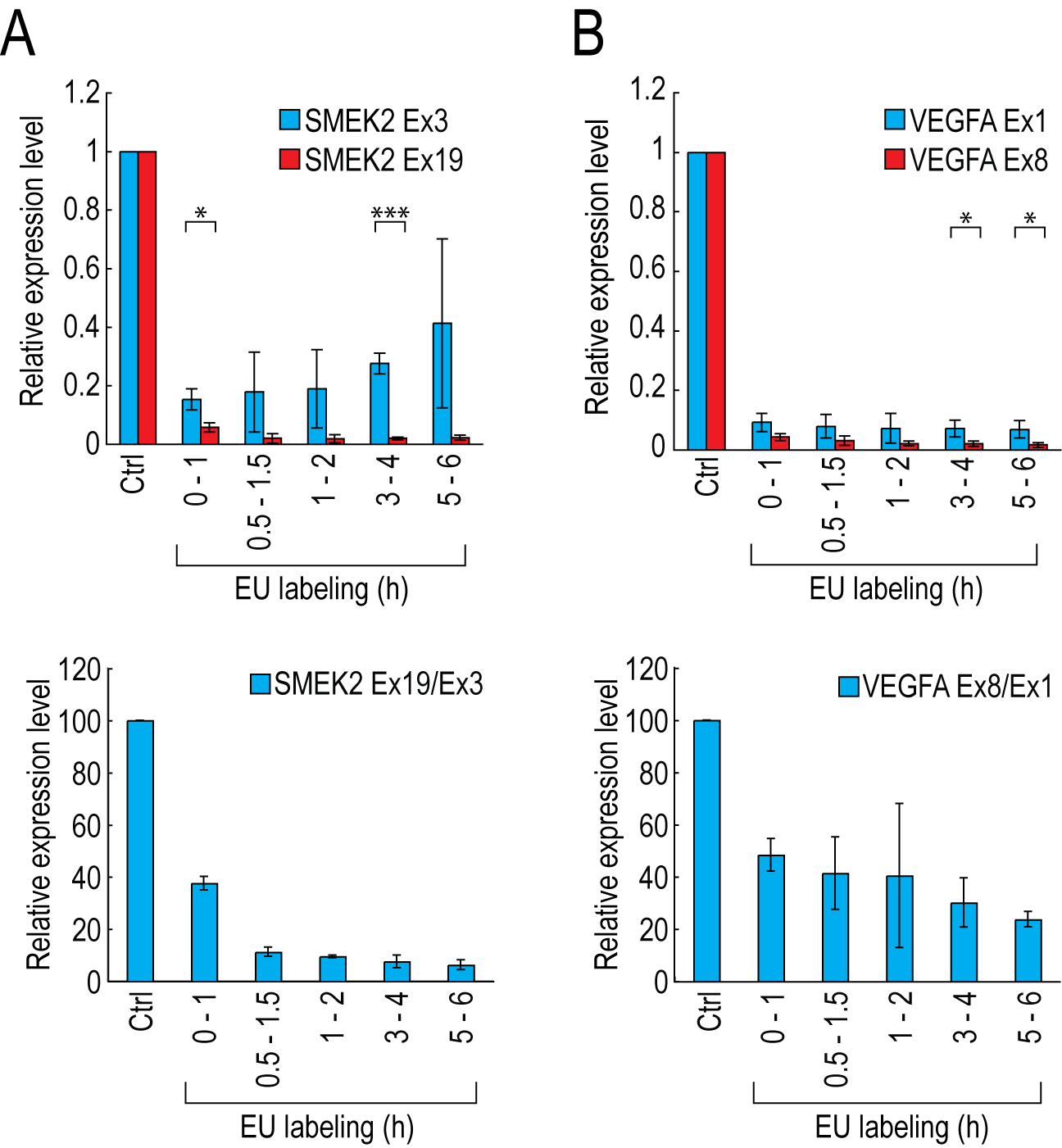

Supplement: Figure S3 — SSA treatment causes 3′-end down-regulation in a time-dependent manner. HeLa cells were treated with 100 ng/ml of SSA, and RNAs were labeled during transcription with EU for 1 hour, as in Figure 2D. Labeled RNAs were analyzed by quantitative RT-PCR to measure the levels of the 5′ ends (SMEK2 Ex3 and VEGFA Ex1) and 3′ ends (SMEK2 Ex19 and VEGFA Ex8) of these genes (upper panels). The ratio between the 3′ and 5′ levels was calculated for each gene (non-treated cells [Ctrl] = 100%) (lower panels). Error bars indicate s.d. (n = 3). Statistical significance was investigated by the t-test (*: p<0.05; **: p<0.01; ***: p<0.001). (TIF) [file pone.0098015.s003.tif]

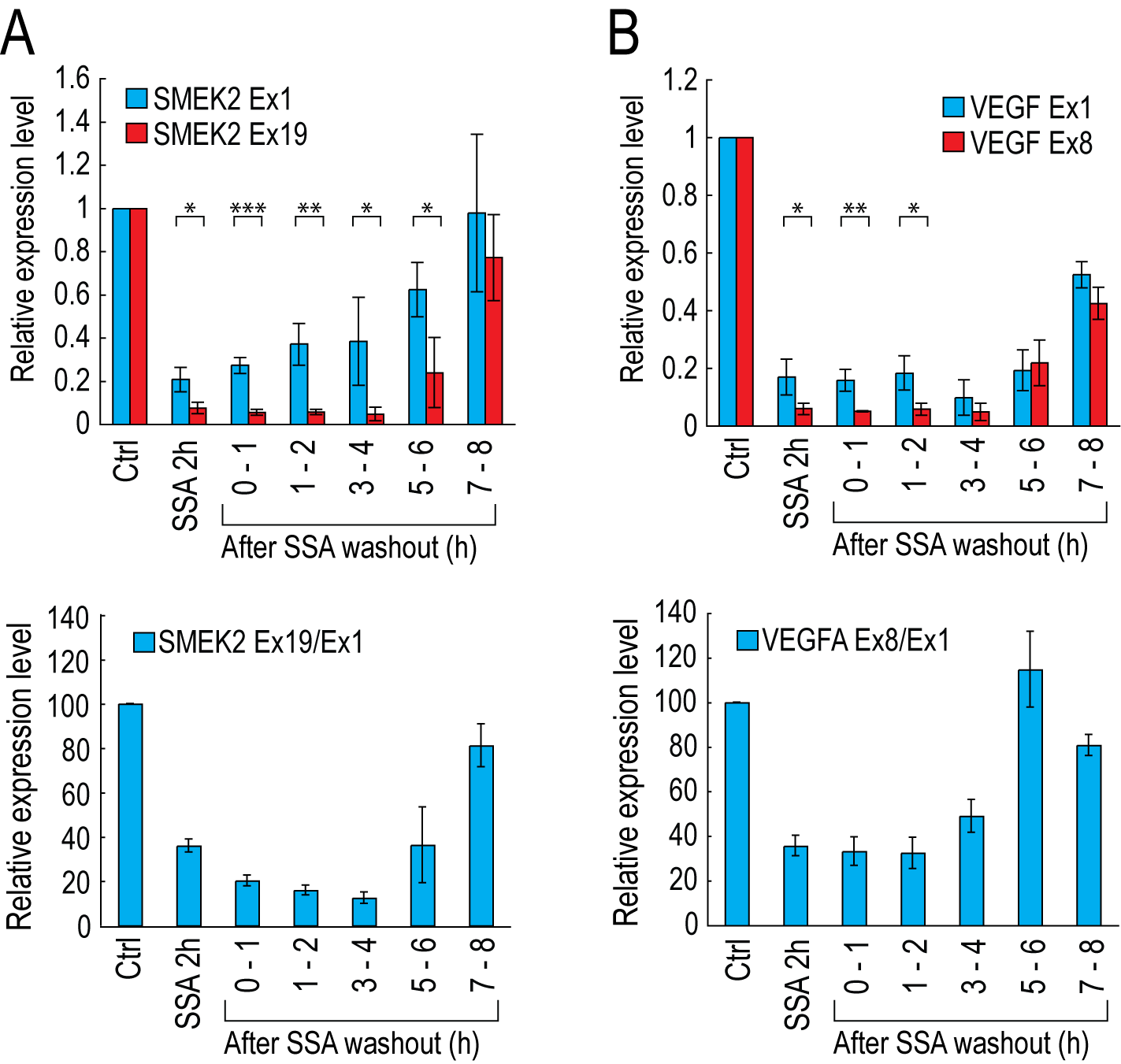

Supplement: Figure S4 — Expression of the 3′ ends of genes recovers after SSA washout. HeLa cells were treated with 100 ng/ml of SSA for 2 hours and washed twice with fresh medium. Cells were cultivated in fresh medium, and RNAs were labeled during transcription with EU for 1 hour, as in Figure 2G. Labeled RNAs were analyzed by quantitative RT-PCR to measure the levels of the 5′ ends (SMEK2 Ex1 and VEGFA Ex1) and 3′ ends (SMEK2 Ex19 and VEGFA Ex8) of these genes (upper panels). The ratio between the 3′ and 5′ levels was calculated for each gene (non-treated cells [Ctrl] = 100%) (lower panels). Error bars indicate s.d. (n = 3). Statistical significance was investigated by the t-test (*: p<0.05; **: p<0.01; ***: p<0.001). (TIF) [file pone.0098015.s004.tif]

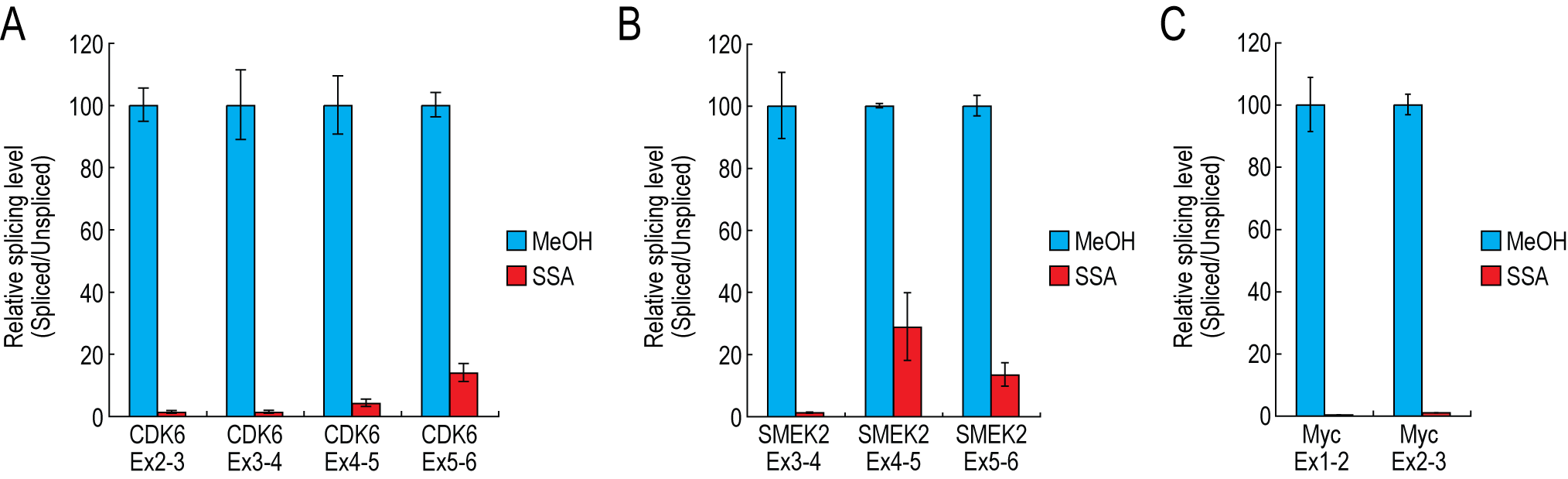

Supplement: Figure S5 — SSA treatment causes global splicing inhibition. HeLa cells were treated with 100 ng/ml of SSA for 4 hours, and RNAs were labeled during transcription with EU between 3 and 4 hours after the addition of SSA. Labeled RNAs were analyzed by quantitative RT-PCR to measure the amounts of spliced and unspliced mRNA. Relative splicing activity was defined as the ratio of spliced to unspliced mRNA (methanol-treated cells [Ctrl] = 100%). Error bars indicate s.d. (n = 3). (TIF) [file pone.0098015.s005.tif]

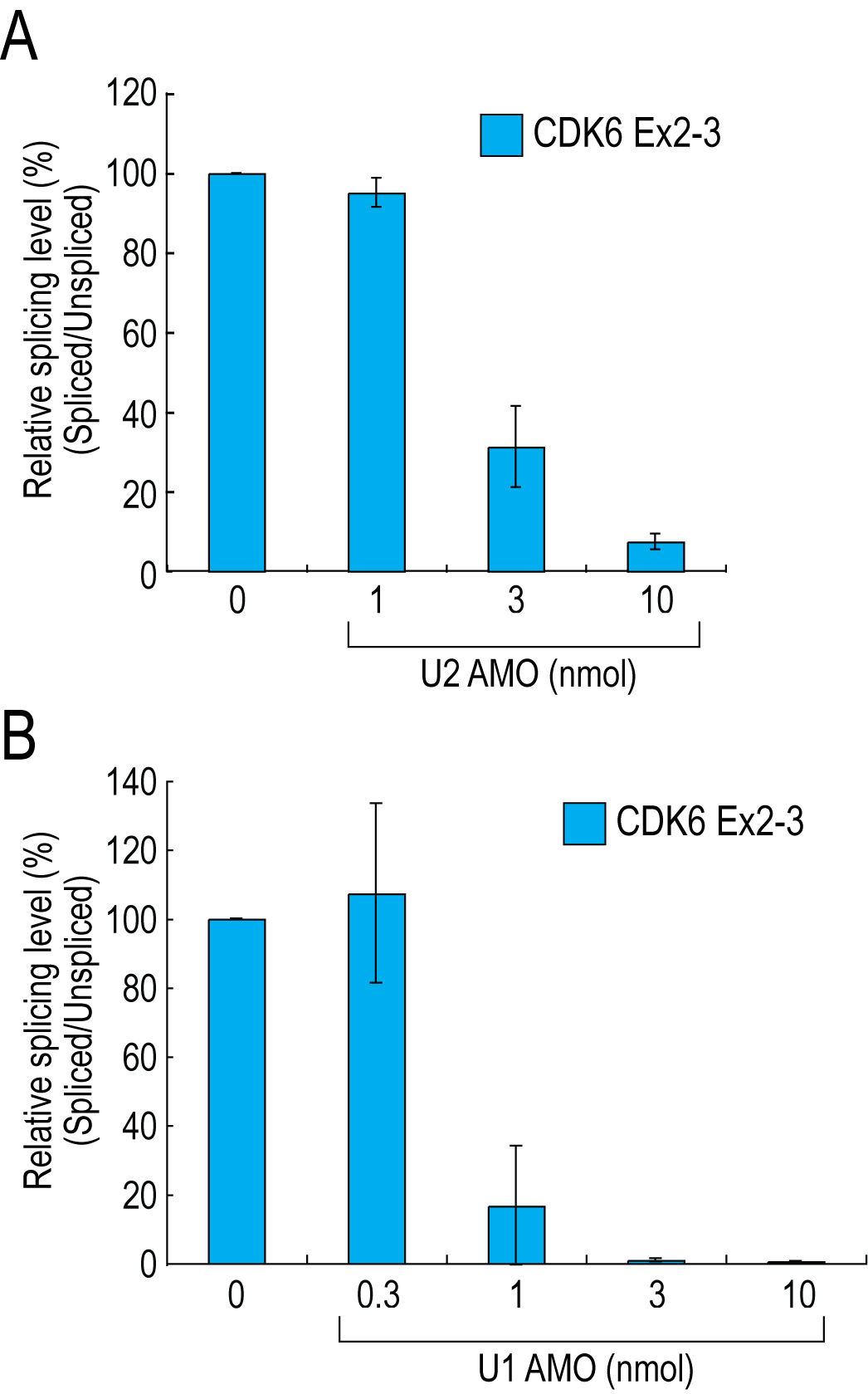

Supplement: Figure S6 — AMO treatment causes splicing inhibition. HeLa cells were transfected with varying concentrations of U2 AMO (A) or U1 AMO (B) as indicated, and then cultured for 6 hours after transfection. RNAs were labeled during transcription with EU between 5 and 6 hours after transfection, and labeled RNAs were analyzed by quantitative RT-PCR to measure the amounts of spliced and unspliced mRNA. Relative splicing activity was defined as the ratio of spliced to unspliced mRNA (methanol-treated cells [Ctrl] = 100%). Error bars indicate s.d. (n = 3). (TIF) [file pone.0098015.s006.tif]

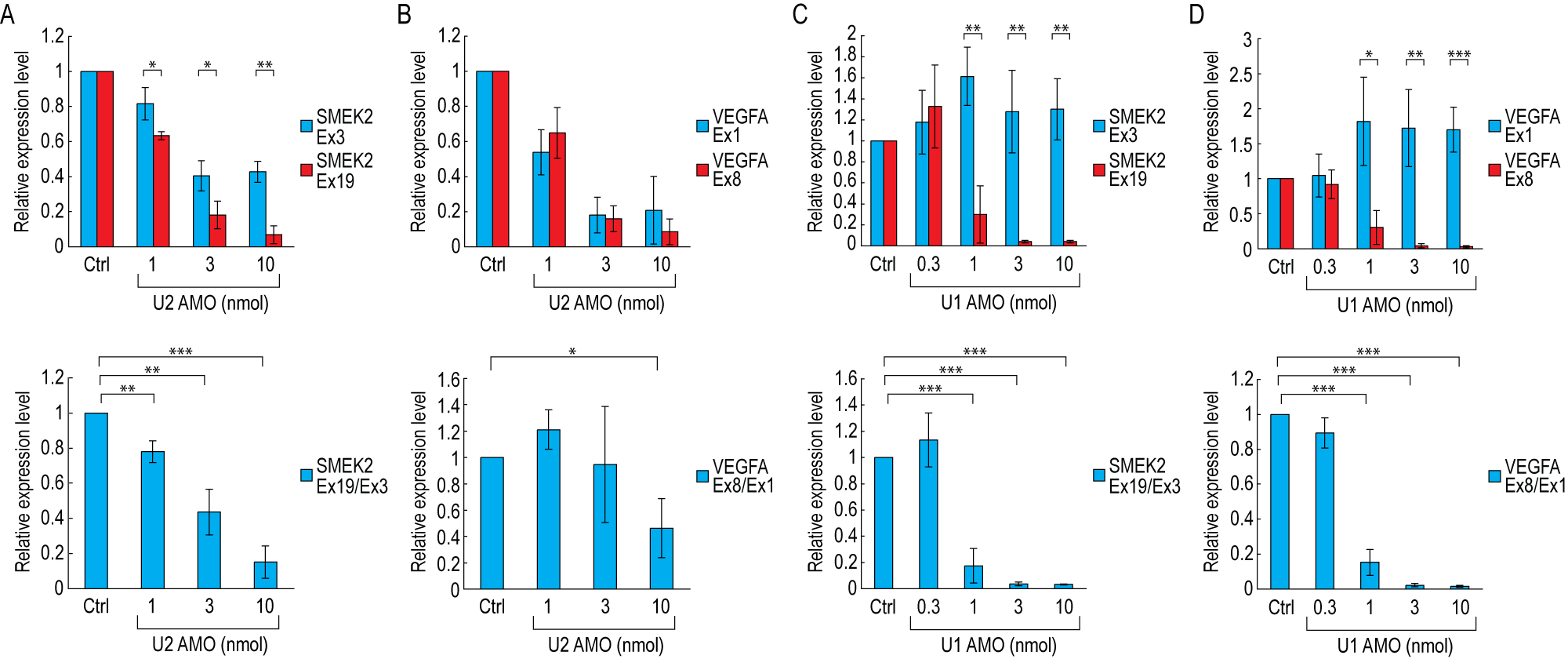

Supplement: Figure S7 — AMO treatment causes splicing inhibition. HeLa cells were transfected with varying concentrations of U2 AMO (A, B) or U1 AMO (C, D) as indicated, and then cultured for 6 hours after transfection. RNAs were labeled during transcription with EU between 5 and 6 hours after transfection, and labeled RNAs were analyzed by quantitative RT-PCR to measure the levels of the 5′ ends (SMEK2 Ex3 and VEGFA Ex1) and 3′ ends (SMEK2 Ex19 and VEGFA Ex8) of these genes (upper panels). The ratio between the 3′ and 5′ levels was calculated for each gene (non-treated cells [Ctrl] = 100%) (lower panels). Error bars indicate s.d. (n = 3). Statistical significance was investigated by the t-test (*: p<0.05; **: p<0.01; ***: p<0.001). (TIF) [file pone.0098015.s007.tif]

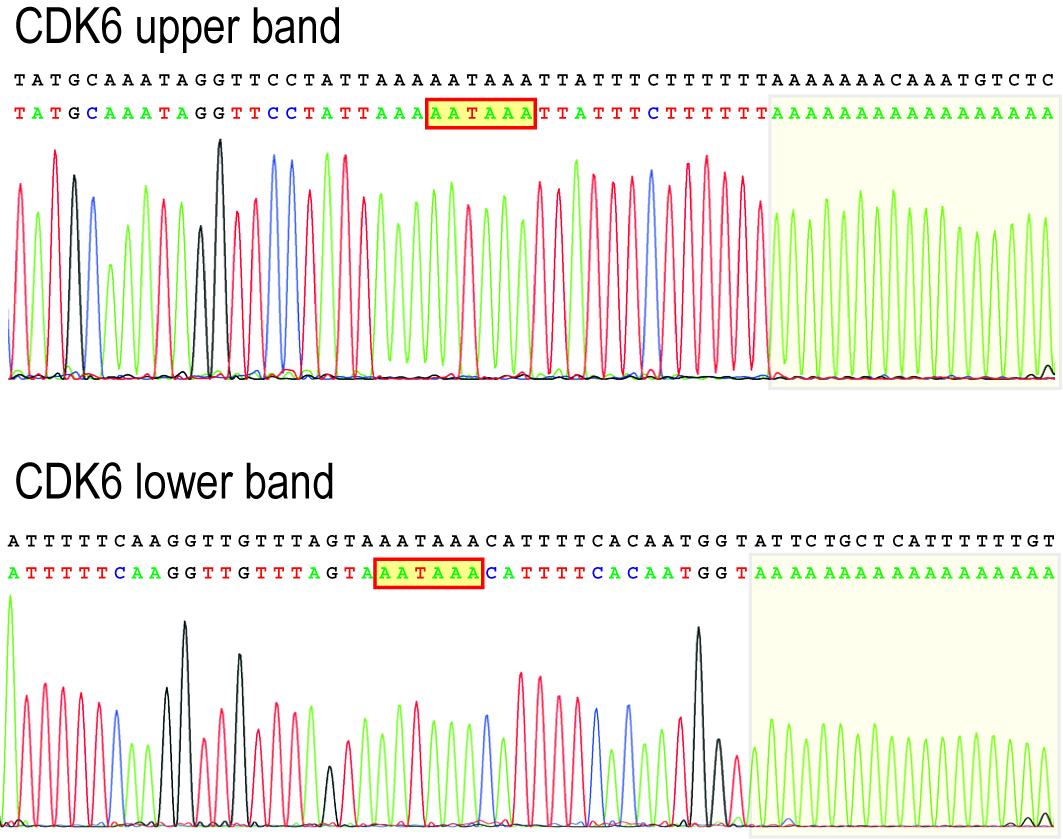

Supplement: Figure S8 — Sequencing of the 3′-RACE products. Sequences of the 3′-RACE products for the upper and lower bands of the CDK6 gene are shown along with the corresponding genomic sequences (in black). The poly(A) tails are shaded, and the putative PASs are indicated by red outlines. (TIF) [file pone.0098015.s008.tif]
