# Supplementary material for: U2 snRNP Is Required for Expression of the 3′ End of Genes
Source: PLoS One. 2014 May 20;9(5):e98015. doi: 10.1371/journal.pone.0098015 (PMC4028248; doi:10.1371/journal.pone.0098015)
Supplement: Table S3 — List of primers used in this study. (DOCX) [file pone.0098015.s011.docx]

Table S3. List of primers used in this study.

For ChIP analysis.

| Name | sequence (5'-3') |
| --- | --- |
| CDK6-A-F | AGAGAGTGCTGGTAACTCCTTCC |
| CDK6-A-R | TGCGAGTGTCAGTCGGCTCT |
| CDK6-B-F | TAGCGATGGAGGCTTCGTAAAGGA |
| CDK6-B-R | AAGTGCAATCAGACAGCCCAGAAG |
| CDK6-C-F | TTACTGTGGAAGGCTCACTGACCAGA |
| CDK6-C-R | ACTCAACACCCTTGGCCAGTTT |
| CDK6-D-F | TGAAAGTGTGGGAGTGGAAAGGCA |
| CDK6-D-R | TTGAGCATTTGCTATGCTGGACGC |
| CDK6-E-F | TTAAGCTGATCCTGCGGAGAACAC |
| CDK6-E-R | TTTCCTTGGAGAAGCAGAGCCTGT |
| SMEK2-A-F | TACTCCGGTTCAAGGCTACGTACT |
| SMEK2-A-R | ATGGCACCAGTATGTGACGTTCCT |
| SMEK2-B-F | AAAGAGACGGGAGAGTGGGTGTAT |
| SMEK2-B-R | TTCACCATGGCTCCAAAGGTTCAG |
| SMEK2-C-F | AGTGGAAGTCACACAGGACCTCAT |
| SMEK2-C-R | TCACATGTGGGCAGGTCAATCAGA |
| SMEK2-D-F | GGAGCAGATTTGGTTATGGCAACATGG |
| SMEK2-D-R | CCCTTTCTGCTTGGATAGAAACAATGAC |
| SMEK2-E-F | GGCTTCCCAGGAACTTGATTGCTT |
| SMEK2-E-R | AAGCTGTCTCCCAGGTTCTGGTTACT |
| EGFR-A-F | GTCCAGTATTGATCGGGAGAG |
| EGFR-A-R | AGACACGCCCTTACCTTTCT |
| EGFR-B-F | GAGAGCTGAACACCTGAAGAC |
| EGFR-B-R | CTCATCACAAACCCTCTTCTCTC |
| EGFR-C-F | TACCCAACAACCAGCCTACTGACA |
| EGFR-C-R | AAAGGTGAGAGGTGGGTCTGACTT |
| EGFR-D-F | GCCTCCACTGCTAAAGTCCACATA |
| EGFR-D-R | ACTTGTGGGATGCTCCAAGCTCAT |
| C-Myc-A-F | GGAGGGATCGCGCTGAGTA |
| C-Myc-A-R | AGGCCTTTGCCGCAAAC |
| C-Myc-B-F | TGCCCCTCAACGTTAGCTTC |
| C-Myc-B-R | GGCTGCACCGAGTCGTAGTC |
| C-Myc-C-F | GTCCAAAGCCTCATTAAGTCTTAGGTA |
| C-Myc-C-R | CAACTTCCCAGGATAGGACATTG |
| C-Myc-D-F | CCTGAGCAATCACCTATGAACTTG |
| C-Myc-D-R | CAAGGTTGTGAGGTTGCATTTG |

For qPCR

| Name | sequence (5'-3') |
| --- | --- |
| 5S for | CGGCCATACCACCCTGAAC |
| 5S rev | GCGGTCTCCCATCCAAGTAC |
| CDK6 Ex2 for | AGTACGAATGCGTGGCGGAGAT |
| CDK6 Ex2 rev | AAACGGCCTCCGTTCTTCAAGT |
| CDK6 Ex3 for | GAACAGACAGAGAAACCAAACTAAC |
| CDK6 Ex3 rev | TTTCAGTGGGCACTCCAG |
| CDK6 Ex4 for | GAGTAGTGCATCGCGATCTAAA |
| CDK6 Ex4 rev | GAGGTTAGAGCCATCTGGAAAC |
| CDK6 Ex5 for | CGTCACGCTGTGGTACA |
| CDK6 Ex5 rev | CAGCCAACACTCCAGAGAT |
| CDK6 Ex8 for | CTGCTGACCAATTGTGCTGCCATT |
| CDK6 Ex8 rev | CACACACACATGCACACACACACT |
| CDK6 Ex2-3 spliced for | ATGCCGCTCTCCACCAT |
| CDK6 Ex2-3 spliced rev | TCCCTGGCTCACCTGAC |
| CDK6 Ex3-4 spliced for | CTTGGATAAAGTTCCAGAGCCT |
| CDK6 Ex3-4 spliced rev | TTTAGATCGCGATGCACTACTC |
| CDK6 Ex4-5 spliced for | AGTTTCCAGATGGCTCTAACC |
| CDK6 Ex4-5 spliced rev | CAAATATGCAGCCAACACTCC |
| CDK6 Ex5-6 spliced for | TCTTGCTCCAGTCCAGCTA |
| CDK6 Ex5-6 spliced rev | TCAACATCTGAACTTCCACGAA |
| CDK6 Ex2-3 unspliced for | TGCAGCTGTGCAACTTAGA |
| CDK6 Ex2-3 unspliced rev | GTTGGCTTATCCTGTCCCTAAA |
| CDK6 Ex3-4 unspliced for | CAAGACTTGACCACTTACTTGGA |
| CDK6 Ex3-4 unspliced rev | CGACTGCCTGATAAGACATGAA |
| CDK6 Ex4-5 unspliced for | TCCAGATGGCTCTAACCTCA |
| CDK6 Ex4-5 unspliced rev | AGACATGGAAGAGGGACAGA |
| CDK6 Ex5-6 unspliced for | CTGGAGTGTTGGCTGCATATT |
| CDK6 Ex5-6 unspliced rev | GCTGCCACTCAAATTCACAAAG |
| SMEK2 Ex3 for | TGCCTTTACCGTCTCCTAAGAGTG |
| SMEK2 Ex3 rev | ATTGCCGGTCTTCGTTCAGGGTAT |
| SMEK2 Ex5 for | TGTTTGGTCAGAAGCAGAGAA |
| SMEK2 Ex5 rev | TTTCCCAGATCTCATCACAGC |
| SMEK2 Ex6 for | CTTAGTTACCTCAGTGCTCTCC |
| SMEK2 Ex6 rev | GCTTGGAACAGCTGCAATAG |
| SMEK2 Ex19 for | GGAAGTTTGGTTGGCTTAGTGG |
| SMEK2 Ex19 rev | GCTCACTGAACAGTTGCAGCATTG |
| SMEK2 Ex3-4 spliced for | ACTTACGTGGAGGAGCTCAA |
| SMEK2 Ex3-4 spliced rev | CTGTTGTTTCTGATATGCAGTATTTGG |
| SMEK2 Ex4-5 spliced for | CTGCATATCAGAAACAACAGGATAC |
| SMEK2 Ex4-5 spliced rev | TCTCATCACAGCCAGCTTTC |
| SMEK2 Ex5-6 spliced for | TGTTTGGTCAGAAGCAGAGAA |
| SMEK2 Ex5-6 spliced rev | ACTTCCACTGATGGGTCTTTAC |
| SMEK2 Ex3-4 unspliced for | CTTACGTGGAGGAGCTCAAG |
| SMEK2 Ex3-4 unspliced rev | CCCAACCAGAGGCACTATC |
| SMEK2 Ex4-5 unspliced for | CCCTATTCTACAGTTCCGATAACC |
| SMEK2 Ex4-5 unspliced rev | AGTTCTCTGCTTCTGACCAAAC |
| SMEK2 Ex5-6 unspliced for | AGAAAGCTGGCTGTGATGAG |
| SMEK2 Ex5-6 unspliced rev | TCGCCACATCCTAATATGTTTGA |
| C-MYC Ex1 for | GGAGGCTATTCTGCCCATTT |
| C-MYC Ex1 rev | CTAAGCAGCTGCAAGGAGAG |
| C-MYC Ex2 for | GGTGCAGCCGTATTTCTACT |
| C-MYC Ex2 rev | AGCAGCTCGAATTTCTTCCA |
| C-MYC Ex3 for | CATACATCCTGTCCGTCCAAG |
| C-MYC Ex3 rev | GAGTTCCGTAGCTGTTCAAGT |
| C-MYC Ex1-2 spliced for | CTTCTCTGAAAGGCTCTCCTTG |
| C-MYC Ex1-2 spliced rev | GTCGAGGTCATAGTTCCTGTTG |
| C-MYC Ex2-3 spliced for | TCGGATTCTCTGCTCTCCT |
| C-MYC Ex2-3 spliced rev | CCTCATCTTCTTGTTCCTCCTC |
| C-MYC Ex1-2 unspliced for | CTTCTCTGAAAGGCTCTCCTTG |
| C-MYC Ex1-2 unspliced rev | AAGTGGACTTCGGTGCTTAC |
| C-MYC Ex2-3 unspliced for | CTGGTGCTCCATGAGGAGA |
| C-MYC Ex2-3 unspliced rev | TATCCAGCCGCCCACTT |
| VEGF Ex1 for | GAGGCGCAGCGGTTAGGT |
| VEGF Ex1 rev | CGGATCAATGAATATCAAATTCCA |
| VEGF Ex8 for | CTGGCGCTGAGCCTCTCTAC |
| VEGF Ex8 rev | CCGGTGTCCTCATCCCTGTA |
| EGFR Ex1 for | CCTGACTCCGTCCAGTATTGAT |
| EGFR Ex1 rev | TCCTCCAGAGCCCGACT |
| EGFR Ex28 for | CTCTGTGCAGAATCCTGTCTATC |
| EGFR Ex28 rev | GGCTGGACAGTGTTGAGATAC |

For 3’ RACE

| Name | sequence (5'-3') |
| --- | --- |
| Oligo dT18-XbaKpnBam | CTGATCTAGAGGTACCGGATCCTTTTTTTTTTTTTTTTTT |
| XbaKpnBam | CTGATCTAGAGGTACCGGATCC |
| NR3C1 Ex2-1 | GCCAAGGATCTGGAGATGAC |
| NR3C1 Ex2-2 | CTCTGAACTTCCCTGGTCGAAC |
| STK17A int1-1 | CACCATGCCTGGCTTCAACTG |
| STK17A Ex2-1 | GAACTAGCACAAGACAATCCTTGG |
| CDK6 int2-1 for | GGAGTATCCAGTTAAGGGTGC |
| CDK6 int2-2 for | CGTAGAGGTACTACAGAGGCAC |
